# Supplementary material for: Are carbon emissions trading and green financial instruments synergistic? -Comprehensive quantitative research based on content analysis
Source: PLoS One. 2024 Mar 7;19(3):e0298601. doi: 10.1371/journal.pone.0298601 (PMC10919601; doi:10.1371/journal.pone.0298601)
Supplement: S1 Data — (ZIP) [file pone.0298601.s001.zip › Data/地级市Z_F工作报告.zip!/╣π╢1⁄2/╒┐╜¡╩╨/└┤╫╘╣1⁄2╓┌║┼í╛┬φ┐╦╩2╛▌═°í┐_2014.docx]

各位代表：

　　我代表市人民政府向大会报告工作，请予审议，并请市政协委员和其他列席同志提出意见。

2013年工作回顾

　　2013年，在市委正确领导下，在市人大、市政协监督支持下，市政府认真落实党的十八大和十八届三中全会精神，按照省委、省政府加快粤东西北振兴发展战略部署，围绕“推动五年崛起、建设幸福湛江”和迈进广东经济发展第二梯队的总目标，面对复杂形势不困惑，面对繁重任务不退却，面对人民期待不浮躁，奋发图强、团结实干、攻坚克难，实现了经济社会平稳较快发展，完成了市十三届人大三次会议确定的年度主要目标任务。

　　过去一年，我们全力打基础谋长远，强产业调结构，抓扩容重提质，惠民生促和谐，经济社会发展实现历史性突破。全市生产总值、金融机构存款余额双双突破两千亿元，消费品零售总额突破一千亿元，固定资产投资近八百亿元，地方公共财政预算收入突破一百亿元，农民人均纯收入突破一万元，主要经济指标实现了两位数增长；湛江钢铁基地全面动工，东海岛掀起建设高潮；城市环境整洁有序，城市面貌焕然一新；“治水”取得新突破，南柳河河水从黑臭变清澈，赤坎水库从脏乱变美丽；科学防御“温比亚”、“尤特”等6次台风袭击，取得吴川“8?15”抗洪抢险全面胜利；成功处置“1?13”管道原油泄漏事件；“湛江海洋周”盛况空前，东盟华商交流会成功举办，开放、生态、奋进的湛江吸引了世界越来越多的目光！

　　一年来，我们主要抓了以下工作：

　　着力加快发展，综合实力迈上新台阶。全市生产总值2060亿元，增长12%，经济总量在全省排名从第九位上升到第八位。人均生产总值2.9万元，增长11.2%。地方公共财政预算收入105.9亿元，增长15%；来源于湛江财政总收入573.4亿元。城镇居民人均可支配收入22371元，增长10.6%；农民人均纯收入10737元，增长12.3%。各位代表，湛江经济总量从百亿元跨入千亿元用了17年，从千亿元跨入两千亿元用了5年，标志着在新世纪第二个十年，湛江迎来了发展机遇期，进入了快速成长期，走进了崛起新时代。

　　着力做大产业，振兴发展增添新后劲。重点项目建设提速，湛江钢铁基地全面动工，完成投资52亿元，焦炉、烧结、高炉、炼钢、连铸、热轧和冷轧七大主体及附属配套工程开工建设，建成30万吨原料码头；“宝钢梦想起航”展览圆满成功。中科炼化项目基本完成红线内搬迁清表和厂区场平，具备全面开工条件。湛江晨鸣一期实现产值78亿元，二期18万吨纸杯原纸、三期19万吨高档文化用纸项目相继动工。全市重点项目完成投资370.3亿元，增长42.2%。固定资产投资795.6亿元，增长39%。工业经济较快增长，规模以上工业总产值2025.9亿元、增长18.2%，增加值659.2亿元、增长14.9%。扎实开展“三服务”活动，建成威立电缆、德利化油器、半岛科技、华思科技、华海结晶果糖等项目，中海油80万吨重交沥青技改项目动工，15万吨中能酒精燃料乙醇项目获得国家核准。实施骨干企业培育行动计划，新增销售收入亿元以上企业16家，其中10亿元以上骨干企业1家。园区带动作用显现，深入开展“三讲三评”，赤坎、廉江、霞山分别获得第一、二、三名。湛江产业转移园获得省扶持资金2.5亿元。10个工业园区新开工项目50个，建成项目24个，实现产值948亿元、增长31.9%。5县（市）生产总值全部突破百亿元，其中廉江突破300亿元。创新发展能力增强，专利申请量、授权量分别增长29.2%和20.6%，成为国家知识产权试点城市；获得国家科技成果奖励1项，新增高新技术企业4家、创新型企业两家；落实校地联席会议制度，新增两个产业技术协同创新战略联盟。新增欢乐家、半球两件中国驰名商标，同德药业设立国家岭南药用芳香油重点研究室。服务业持续发展，服务业增加值824.2亿元、增长13.2%，社会消费品零售总额1010.7亿元、增长15%。港口吞吐量1.8亿吨，增长5.4%。民航旅客吞吐量创历史新高，达到70.5万人次，增长36.4%。银信物流一期建成，北部湾农产品流通综合示范园首期基本完工，举办全国农产品流通跨区域投资促进与品牌推介活动；海田物流产业园挂牌，东盟城动工。光大银行等金融机构落户，人民大道“金融街”基本形成；金融机构本外币存款余额2173.4亿元，金融存贷比56.5%。评选产生湛江新八景，接待游客2543.5万人次、旅游总收入155.5亿元，分别增长13.6%和22.3%。现代农业稳健发展，农业增加值421.4亿元、增长6.1%。新增农民专业合作社288家。建成基本农田保护示范区4.96万亩；雷州东西洋水稻良种覆盖、机耕机收和病虫综防率接近100%。建成农垦沃尔多曾祖代和正大父母代种猪场。海洋产业总产值1308.4亿元，增长12.6%。湛江特色水海产业国家农业科技园获得批准，国家级海产品质量监督检验中心建成使用。建成小型农田水利示范镇7个，完成41宗小型水库除险加固工程。

　　着力扩容提质，城市建设取得新进展。编制完成湛江城市发展战略规划、城市总体规划和近期建设规划。新区建设全面启动，海东新区总体规划获得省政府批准，完成征地9400亩，海东快线、恒大绿洲等项目和岸线滩涂综合整治工程动工。西城新区规划通过评审。中心城区提质步伐加快，城市原点广场、西澜湾渔人码头、中央商务区和海上运动中心动工；海滨大道完成改扩建，湖光快线、社坛路建成通车，广州湾大道动工建设。完成3个“水浸街”整治和康顺路、康宁路等8条市区道路“白改黑”，实施117条小街小巷改造，“三旧”改造取得新突破。渔港公园海滨浴场建成开放。城区公交实现WIFI全覆盖。建成市区生活垃圾处理场三期，垃圾焚烧发电厂动工，城市生活垃圾无害化处理率100%。

　　着力改善交通，基础建设迈出新步伐。深湛高铁茂湛段建成通车，完成湛江机场、火车西站升级改造。完成广湛高速官渡出入口立交和90.8公里省道路面改造，基本建成东海岛疏港公路和省道285线吴川龙首至市区段，基本完成环半岛一级公路徐闻段10公里征拆清表和路基工程。湛江港霞山港区30万吨级散货码头竣工，宝满港区80万标箱集装箱码头投入使用。鉴江供水枢纽工程建成通水。电网建设完成投资10亿元，建成6个110千伏输变电工程。

　　着力加大“治水”，生态建设取得新成效。“创卫”通过国家暗访，城市洁净水平明显改观，社会各界好评如潮。“治水”取得重要成果，南柳河整治完成6.3公里河道截污清淤和生态护坡建设；完成赤坎水库综合整治，建成7.2公里环湖绿道，瑞云湖生态公园建成开放；鹤地水库环境综合整治成效初显。城市饮用水源和近岸海域水质达标率100%，环境空气质量保持全国环保重点城市前列。廉江开发区、石角两间污水处理厂投入运行，东海岛、坡头两间污水处理厂基本建成，城镇生活污水处理率86.7%。完成造林20.2万亩，森林覆盖率29.5%。开展生态文明区城镇村创建行动，新增国家级生态镇3个。

　　着力改革开放，市场主体焕发新活力。体制改革深入推进，公布实施第一批行政审批改革事项目录，取消、调整行政审批事项189项，取消、免征和降低收费138项。初步理顺市、区两级事权财权。发行首支城投债——基投集团12亿元企业债券。商事登记制度改革全面启动，新增私营企业4603户、个体工商户28382户，分别增长80%和40.3%。民营经济提速发展，深入实施民营企业“十百千”投资计划及“乡贤反哺工程”，引进项目60个、总投资677亿元，建成项目19个；民间投资484.7亿元、增长53.2%，民营经济增加值1250亿元、增长14%。保利、万达、恒大、荣盛等著名大型企业进驻湛江。外经贸快速增长，举办湛江（香港）投资说明会，东海岛、宝满港区口岸开放取得重大进展，湛江国联在美国暖水虾反补贴调查案中胜诉。外贸进出口总额55.1亿美元、增长17.3%，其中出口26.3亿美元、增长18.7%；实际吸收外资1.3亿美元、增长51%。开放政府建设向前推进，建成行政服务中心办事大厅和省网上办事大厅湛江分厅，政务服务事项实现网上全程办理；开通12345市民服务热线，建立局长接听群众来电制度。34个市直单位440项行政职权目录全面公示，实行部门承诺制取得良好效果。完成政府门户网站升级改版，开通湛江政务微博群，举行政府新闻发布会20次，市政府领导轮流在线访谈12次。与澳大利亚杰尔顿缔结友好城市，国际友好城市达到6个。

　　着力改善民生，百姓福祉获得新提升。市财政社会民生投入177.4亿元，增支20.5亿元，占公共财政支出70.2%。民生保障有效加强，较好完成15件民生实事，建成保障房3285套，完成1万户农村困难家庭住房改造和20条贫困村640户整村搬迁，建成水库移民新村85条，1056户台风“全倒户”全部搬进新居。新增城镇就业7.3万人，农村劳动力转移就业8.6万人。全面发行社会保障卡，实现医保异地就医住院费用即时结算，城乡居民大病保险经验在全国推广；企业退休人员基本养老金每人每月达到1618元、增加172元，城乡低保补差水平分别提高到每人每月242元、111元。发放“两低群体”价格调节基金补贴6200万元。村村通自来水工程深入实施，104条行政村、14.1万人用上了自来水。创建基本公共服务均等化示范村50条，村级公共服务中心受到群众欢迎。新一轮扶贫开发“双到”工作全面启动。社会事业不断进步，创建教育强区2个、强镇（街）35个；“名校长、名教师”培养工程和千名教师交流支教、万名教师学历提升计划启动实施；清理整治无证幼儿园615所，学前三年入园率90.7%；市四中新校区建成使用；广东海洋大学、广东医学院获得博士学位授予权。实现免费孕前优生健康检查全覆盖，人口计生工作取得新进步；市120医疗急救指挥中心动工；湛江中心人民医院新院主体工程完成投资50%；改造薄弱乡镇卫生院10间。建成平价粮油店123家、平价药店40家、“阳光厨房”538家，物价工作获全国先进。建成两个区级图书馆和两间特色博物馆，举办第八届湛江艺术节，雷剧成功申报国家非物质文化遗产；完成6258户散居农户、199条出海渔船“户户通”广播电视。市档案馆新馆建成使用，《湛江市志（1979-2000）》出版发行。湛江奥体中心“一场三馆”完成钢结构安装并封顶；举办第十二届市运会，湛江籍运动员在第十二届全运会获得6金5银好成绩。双拥共建、国防教育和妇女儿童、民族宗教以及统计、审计、老区、粮食、人防、气象等工作取得新成绩。民主法治与精神文明建设稳步推进，被评为“全国法治城市创建活动先进单位”。妥善处理判决生效的政府历史债务。办理人大代表建议、政协委员提案307件，行政复议案件结案率100%。开展“一清两禁”专项行动，破获涉枪案件293宗，集中整治社会治安重点地区和突出问题，严重暴力犯罪案件下降15.3%。陈光保、李文波荣获“广东省道德模范”称号和“第四届全国道德模范提名奖”。

　　各位代表，过去一年，我们在改革中发展，在克难中前行，在挑战中奋进，成绩来之不易。这是中共湛江市委正确领导、科学决策的结果，是市人大、市政协有效监督、鼎力支持的结果。成绩凝聚着各级人大代表、政协委员的辛勤付出，凝聚着驻湛部队、驻湛单位和社会各界的参与支持，凝聚着海内外湛江乡贤和友好人士的倾情回报，凝聚着800万人民的智慧汗水。我谨代表市人民政府，向你们表示衷心的感谢，致以崇高的敬意！

　　成绩固然可喜，问题不容回避。主要表现在：发展不够充分，发展速度和质量有待提升；中科炼化项目推进不够快；个别民生实事尚未完成；港口增长乏力，南方大港地位受到明显挑战；投资环境没有明显改善，全民创业氛围不浓；以市场化手段推进工业化、城镇化的办法不多，部门执行力有待提高，干部干事创业的精神需要进一步提振等等。对此，我们将认真对待、切实解决。

　　2014年工作安排

　　今年政府工作的总体要求：全面贯彻党的十八届三中全会和省委十一届三次全会精神，全面落实省委、省政府加快粤东西北振兴发展战略部署和市委十届六次全会精神，坚持好为标准优质增长，快字当先借势发展，稳中求进协调发展，改革创新跨越发展，确保经济发展、城市繁荣、生态良好、社会稳定，确保五年崛起取得重大突破。

　　今年经济社会发展预期目标：生产总值增长12%；工业增加值增长14%；固定资产投资增长25%以上；社会消费品零售总额增长15%；地方公共财政预算收入增长13%；外贸进出口总额增长6%；实际利用外资增长10%；居民消费价格涨幅控制在3.5%左右；人口自然增长率控制在11.5‰以内；城镇登记失业率控制在3.5%以内；市区生活污水处理率94%；单位生产总值能耗下降3.66%。

　　各位代表，完成今年目标任务，我们拥有众多机遇和优势，同时面临重重矛盾和困难，我们难在突出重围任务重，省内及北部湾兄弟城市发展迅猛，湛江陷入区域竞争包围圈，建设中心城市任重道远；难在投入巨大产出小，尽管重大项目陆续动工，但在近期无法形成产能效益，经济增长空间有限，财政运转相当艰难；难在双重考验压力大，作为后发地区，我们既有经济振兴重任，又有生态坚守责任，在奋发崛起的振兴之路上，如何凝聚力量、实现突破，建设美丽富裕的湛江，考验着我们的智慧和能力。挑战与机遇并存，困难大，我们决心更大；困难多，我们办法更多。我们要始终保持清醒的头脑，保持旺盛的实干精神，保持开放的发展势头，坚定信心，乘势而上，扎实抓好以下工作。

　　——加快新型工业化，走转型发展之路。坚定发展工业，突出抓园区、上项目、促发展。抓好120个重点建设项目，其中新开工项目34个，建成项目10个，年度投资402.8亿元。

　　加大工业项目推进力度。落实钢铁、石化、造纸重大项目联席会议制度，保证项目无障碍、封闭式施工。湛江钢铁基地完成投资116.5亿元，启动建设钢铁配套产业园，年底建成自备电厂1号机组。中科炼化项目全面动工，完成投资20亿元以上。湛江晨鸣完成投资21亿元，建成18万吨纸杯原纸项目，启动15万吨生活用纸和70万吨木浆项目前期工作。确保冠豪高新特种纸、双林生物医药等项目达产达效，建成一品家具产业基地、珠江啤酒易拉罐生产线等项目，动工建设大唐电厂、京信电力等项目，加快京能火电前期工作，推进银亿镍合金冶炼、鼎亿大型钾肥等项目。实施500千伏东海岛输变电等项目，完成电网建设投资20亿元。

　　促进产业园区扩能增效。扎实开展“三讲三评”，建好产业园，延长产业链，发展产业群。东海岛园区动工建设鹏尊能源等8个项目，建成王老吉饮料等3个工业项目、东腾路等6条干道和钢铁、石化项目工地配套道路，完成岛东大道路基和石化园区620公顷填海造地工程，加快商务、物流配套设施建设，完善东海岛口岸政务中心查验设施。奋勇高新区建成南国药业等首批5个项目，启动建设高端装备制造园区。廉江园区建成华强电器等10个项目，启动水东烈片区首期开发。吴川园区完成二期基础设施“七通一平”，建成浩信管桩等5个项目。遂溪园区建成尚方舟食品等13个项目，支持岭北工业区建设食品工业园区。规划建设湛江森工产业园，支持奋勇高新区和雷州、遂溪、徐闻、赤坎产业园申报省级产业园区。

　　增强创新驱动能力。推进创新链、产业链和资金链“三链融合”,促进科技金融产业融合发展。突出完善风险投资机制，设立创业投资引导基金和融资担保基金，支持社会资本设立科技孵化基金，建成科技企业孵化器大楼。深化校地合作，推进协同创新，振兴海洋科技，发展海洋产业，壮大海洋经济。做大湛江特色水海产业国家农业科技园，启动100个技术创新重点项目和10项关键技术攻关、10个国家海洋创新发展区域示范基地项目，建设5个产业技术协同创新战略联盟，动工建设湛江工业技术研究院。以湛江湾区为中心，深化政产学研合作，发展钢铁、石化、造纸和家电、医药、水海六大产业集群，打造“南方海谷”。

　　——加快新型城镇化，走升级发展之路。坚持以扩容提质为抓手，打造由中心城区、卫星城和中心镇组成的城镇链。

　　加快城市升级。推进布局升级，实现生产空间集约高效、生活空间宜居适度、生态空间蓝天碧海；推进管理升级，加快城市管理从粗放向精细提升；推进交通升级，规划建设环城高速公路；推进生态升级，整治市区水库、公园、河渠；推进产业升级，加快城区临港重化企业环保搬迁、功能腾退，打造城市发展升级版。

　　支持做大县城和重点中心镇。做大县域经济一靠工业化，二靠城镇化。坚持市县统筹，编制实施县城规划，加快建设雷州城区环城东路、廉江城区北部湾大道、吴川城区滨江路和遂溪城区滨河大道等市政干道，推动县城扩容提质；促进产城融合，推动企业进园、园区近城，支持投融资、土地经营和户籍管理制度改革创新，增强县城集聚发展能力；建成一批污水、垃圾处理设施，提升县城基本公共服务水平。深化垦地合作，把有条件的农垦农场土地纳入乡镇规划，推动徐闻曲界、雷州英利等11个镇，与华海、东方红等13个农垦农场实现镇场融合发展。理顺县镇两级事权财权，有效保障乡镇运转。

　　完善城市规划修编。争取城市总体规划年内获得国务院批复。完成生态控制线划定，编制中心城区扩容提质建设规划，完成城市综合交通、轨道交通、地下空间利用、给排水和消防专项规划编制，实现中心城区控规全覆盖。

　　深入实施“三环四通”。加快交通基础设施建设，大力发展水陆空立体运输。重点推动建设深湛高铁江茂段，加快建设合湛高铁，全面建设东海岛铁路。动工建设海西快线；完成海东快线桥梁施工和环半岛一级公路徐闻段25公里路基、桥涵工程；建成海湾大桥西连接线；推动建设湛徐高速南延长线和吴川大王线公路及蓬吴线二期、三期；启动国道207线、325线路面改造；改革农村公路养护体制，加快县乡道路改造；加快危桥改造步伐，完成廉江石角西桥重建；抓紧环城高速、东海岛至雷州和汕湛高速湛江段前期工作。完成湛江国际机场前期工作，力争获得国家批准；力促民航旅客吞吐量突破100万人次。实施宝满港区4.4公里和东海岛港区1.8公里岸线开发，兴建东海岛港区通用杂货码头；实施徐闻港客滚码头和湛江港30万吨级航道扩建；推动县域渔港、港口建设，壮大县域临港产业。大力发展海湾水上巴士，是方便市民出行、丰富海湾旅游、发展低碳交通的现实需要。编制实施水上巴士规划，统筹开辟航线，加快建设靠泊码头和配套设施，年内完成水上巴士经营主体招投标，启动建造3条近距离航线的船舶和码头，力争2015年春节前新开通一批水上巴士航线，形成多样化城市客运体系。

　　加快海东新区开发。坚持产城融合互动，统筹地上地下空间，加快新区建设步伐，年内完成投资50亿元，建成湛江奥体中心及沿海大道等配套设施和海东观海长廊，开工建设南粤银行金融大厦、奋勇大道南段等19个项目；加快20公里岸线、520公顷滩涂综合整治，启动市区内湾北部海岸线整治；强化海洋湿地保护，建设海东生态公园。加快调顺跨海大桥前期工作，力争年内动工建设。开展湛江湾过海隧道前期工作。

　　提升海西发展质量。加快湛江化工厂、湛江电厂环保迁建工作，加快外贸、霞海码头和湛江港调顺岛港区功能腾退，支持海滨船厂迁建改造。加快建设保利城市原点广场、万达广场和海上运动中心等项目，完成投资72.5亿元。建成霞山法式风情街和赤坎三民路民国风情街。结合海湾西岸整治，新增1个海滨浴场。实施24个“三旧”改造项目，完成投资44.5亿元，开创“三旧”改造新局面。启动人民大道南等交通拥堵路段地下空间开发。加快广州湾大道建设，完成椹川大道北、乐山大道等12条道路“白改黑”，建成海丰路、新湖大道、华信大道等5条道路，动工改造南方路、康强路，动工建设振兴路、海大路和体育北路西段等5条道路。实施麻章湖光海岸线整治，突出科教、交通、生态、宜居功能，加快西城新区建设步伐。

　　各位代表，湛江是我们生活的家园，海湾是我们共同的财富。从湛江港沿海往北，经保利城市原点广场、西澜湾渔人码头、法式风情街、南珠广场、渔港公园海滨浴场，沿中央商务区、海上运动中心、金沙湾海滨浴场、湛江文化中心，再到滨湖公园，是宝贵的33公里黄金岸线。正在建设的广州湾大道是一条景海相映的休闲走廊，是湛江人民多年的梦想、愿望和期待，是还海于城、还岸于民、还景于自然的现实需要，也是企业转型升级、再创辉煌的现实需要。全市人民要顺势而为，共同努力，加强军民团结，加快企业环保搬迁，加快生产功能腾退，让城市拥抱大海、大海环抱城市，湛江重归自然、再现美丽！

　　继续推进“一清四治”。清“地”：完成市区91宗1.6万亩闲置土地清理，分类处置批而未征、征而未供的土地，依法收回未动工开发满两年的闲置土地。治“水”：整治百姓渠等22条市区河渠，完成南柳河综合整治；推动南渡河水系综合整治；加强九洲江流域与鹤地水库水污染区域联防联治，启动鉴江、袂花江流域水患综合整治，启动甘村水库和南溪河上游综合治理；建成奋勇、坡头、东海岛污水处理厂，动工建设赤坎水质净化厂三期和海东新区、屋山、平乐3间污水处理厂，启动南溪河污水处理厂前期工作。治“堵”：坚定整治当月大见成效、整治三月根本好转、整治半年成为习惯的目标，打赢市区交通秩序整治攻坚战；加快实施拥堵十字路口立体交通改造，建成交警智能指挥中心，开辟公交专用通道，建设畅通文明城市。治“乱”：开展“城市精细化管理年”活动，深入整治“六乱”，查处影响市容市貌、破坏环境卫生、毁坏城市绿化行为。治“违”：加强联合执法，落实巡查监控、拆除管理等措施，用铁的手腕、钢的意志严厉打击市区违法建设、违法用地和违法占用岸线、海域滩涂行为。

　　提升服务业发展水平。坚持以中央商务区、海东新区为核心，以金融、商务、中介、科技和总部经济为主要业态，构建服务业集聚区，打造金沙湾及人民大道北高端商贸圈。提升信息化水平，建设大型信息数据交换平台，拓展电子政务，发展电子商务，建成数字化城市管理中心。开展农产品批发市场升级整治，动工建设北部湾农产品流通综合示范园二期，建成湛江商贸物流城等项目，支持建设廉江塘蓬石材市场；海田物流园区完成投资35亿元，建成国际车城二期等项目。促进房地产市场健康发展。加大城市旅游宣传，唱响“湛蓝的海、湛蓝的天”，编制实施滨海城市旅游规划，创建省滨海旅游示范市。完善城市旅游标识系统，开发高端海上旅游线路，建设智慧旅游电子商务平台和旅游自助服务驿站。动工建设南三岛滨海旅游示范区环岛公路、休闲渔港旅游区等项目。创建湖光岩5A级景区。

　　——加快农业现代化，走统筹发展之路。贯彻中央1号文件，全面深化农村改革，着力强农业、惠农村、富农民，让农业成为致富产业，农民成为体面职业，农村成为安居乐园。构建新型农业经营体系，鼓励承包经营权向专业大户、家庭农场、农民合作社、农业龙头企业流转，发展农业规模经营。加快现代农业标准化示范区建设，重点建设雷州东西洋22万亩粮食生产基地，打响东西洋优质大米品牌。建设湛江农垦循环经济示范区，实施正大农业饲料厂、肉鸡加工厂等项目。扩大政策性水稻种植保险覆盖面，开展甘蔗种植保险试点。加强基层农技推广服务，提升现代农业装备水平。改良甘蔗品种，推进糖业种植、设备、技术、管理和产品升级。加快渔船更新改造，组建远洋捕捞船队；支持水海产业转型升级，发展现代、休闲渔业。实施“南珠”振兴工程。加快雷州青年运河灌区节水改造，完成鉴江供水枢纽配套工程，实施红星、合流水库扩容，完成小（2）型水库除险加固工程60宗。完成850公里农村公路硬底化改造。

　　——加快推进生态化，走持续发展之路。发挥生态优势，坚持生态发展，建设生态文明，是湛江实现新型工业化、城镇化的必然选择，必须把生态文明建设贯穿经济社会发展全过程，成为全市人民的自觉行动。

　　发展生态经济。注重点上开发、面上保护，细化主体功能区，逐步取消重点生态地区GDP考核。注重经济发展、生态优先，强化环保约束，实施环境损害依法赔偿、刑事追究制度；禁止生产销售粘土实心砖。注重开发保护并举，加强自然岸线保护。谋划建设海东新区中美生态城和东海岛中欧生态经济合作示范区；建设东海岛钢铁石化循环经济示范区，打造新型工业化产业示范基地。

　　建设生态城市。推进“五城同创”，创建国家卫生城市，“创模”、“创文”取得重大进展。净化空气：整治市区“扬尘”；加快淘汰“黄标车”，设置“黄标车”限行区；建成湛江电厂4号机组脱硝、东兴公司催化裂化装置脱硫设施。每天发布PM2.5空气监测数据。美化环境：建设沿海景观林带200公里，营造碳汇林2.5万亩；规划建设滨湖公园、南国热带花园环园绿道，改造海滨、寸金公园和人民广场。推动市区生活垃圾分类处理，迁建医疗废物处理厂。亮化城市：实施城市景观亮化工程，“十一”前完成市区主要道路、河流、岸线两侧点亮增亮。

　　建设生态乡村。扎实推进生态文明区城镇村创建行动，推进乡村垃圾处理、污水治理和卫生改厕，加快乡村道路硬化、环境绿化、民居美化。开展“最美村庄”、“最美农户”系列评选，建成生态文明村590条。坚持创建生态文明村、建设基本公共服务均等化示范村与新一轮扶贫开发有机结合，切实改变农村面貌。

　　建设生态林。规划建设以县（市）、单位命名的千亩、万亩生态林，年内建设徐闻曲界万亩“热带雨林”首期、南三岛千亩沿海防护林和改造鹤地水库5千亩水源涵养林等生态林示范区，实施三岭山森林公园5千亩造林复绿，湛江农垦和雷州林业局要优化林种，建设规模化生态林，提高生态林覆盖率。

　　各位代表，走向生态文明新时代，建设美丽富裕新湛江，是我们的历史重任。建设生态林,“功成不必在我”,希望在前方，挑战在路上。我们要通过一届又一届政府的不懈努力、一代又一代人的接力传递、一片又一片生态林的建设修复，拓展绿色空间、积累绿色财富、建设绿色家园，实现发展与美丽共赢！

　　——加快投资环境国际化，走开放发展之路

　　深化体制改革。推进市场化改革，处理好政府与市场的关系，注重发挥市场配置资源的决定性作用。完成商事登记制度改革。支持发展混合所有制经济，促进民营企业参股国有资本投资项目。深化国有企业改革，加强国有资本监管，完善国有资本经营预算制度，提高国有资本收益上缴财政比例。组建市公共资源交易中心。加快建设广东（湛江）统筹城乡发展金融改革创新综合试验区，支持湛江港集团上市，支持南粤银行发展，争取1家农信社改制成为农村商业银行。改进预算管理，建立全口径预算编制体系。出台新一批市级行政审批改革事项目录。坚持先行先试，探索东海岛石化园区、海东新区岸线滩涂综合整治海域使用管理“直通车”试点；建设东海岛省级节约用地示范区。理顺南三岛、奋勇高新区和东海岛、海东新区管理体制。

　　改善营商环境。坚持利企安商，抓好“三服务”工作，落实骨干企业培育行动计划，继续改善政务、诚信、治安和交通四大投资环境，降低企业营商成本，破解企业融资难题；提升网上办事大厅和12345市民服务热线效能，建设流程最优、效率最高、服务最好的政务环境。依法打击阻工闹事、抢栽抢种抢建等违法行为。健全社会信用与市场监管体系，建设“信用湛江”。

　　深化开放合作。全力办好中国海洋经济博览会，充分展示湛江建设海洋强市的广阔前景。培育壮大水产品、小家电外贸转型升级示范基地，发展外贸电商和人民币跨境融资业务，加大出口信用保险支持，启动设立欧美、东盟经贸代表处，支持企业开拓市场、扩大进出口。推动构建“湛茂阳”临港经济圈，扩大与珠三角、港澳台、北部湾等区域经济体务实合作，积极参与21世纪海上丝绸之路建设，打造中国远洋门户城市。与更多沿海开放城市建立战略合作伙伴关系，深化国内外友好城市交流合作。

　　加快市场开放。在外商投资、民间投资和工业、商业、基础设施等领域推行负面清单管理，落实“非禁即入”投资政策，确保各类市场主体权利平等、机会平等、规则平等。加强人才队伍建设，落实省扬帆计划重点人才工程项目，引进具有较强创新创业能力的产业领军人才或团队，集聚天下英才，给力湛江振兴。推进实施民营企业“十百千”投资计划和“乡贤反哺工程”，发动海内外华侨、乡贤和商会、社团组织回乡创业，掀起发展在湛江、兴业在湛江、创富在湛江的高潮！

　　各位代表，封闭必然落后，开放才能繁荣。建设现代化城市，必须加快国际化步伐。我们要以大海般的包容胸怀，实行更加积极主动的开放战略，加快建设开放政府、开放城市和开放市场，打开城门天地阔，真诚笑迎八方客，让湛江成为海内外朋友的投资宝地，让一切劳动、知识、技术、管理和资本的活力在红土大地竞相迸发，让一切创造财富的源泉在雷州半岛充分涌流！

　　——加快基本公共服务均等化，走和谐发展之路。坚持多谋民生之利，多办民生实事，多解民生之忧。

　　强化就业与社会保障。坚持以高校毕业生为重点，拓展青年创业就业空间，广辟农村转移劳动力、城镇困难人员、退役军人就业门路。减免行政事业及经营服务性收费，支持全民创业；推动工业园区采用BOT等模式兴建标准厂房，引进中小微企业入驻创业。组织开展职业技能竞赛。新增城镇就业7.5万人、农村劳动力转移就业8万人。推动基本医疗与医疗救助有效衔接，扩大异地就医住院费用即时结算范围，基本医疗保险参保率稳定在98%以上。发展社会养老服务，落实企业职工养老保险扩面征缴。健全社会救助制度，把保障困难群众基本生活的底线兜住兜好。

　　发展社会各项事业。创建教育强县（市、区）3个、镇（街）30个，建设5所县（市）特殊教育学校，建成300所特色文化校园；落实“名校长、名教师”培养工程和万名教师学历提升计划，鼓励城镇教师到农村支教；基本建成湛江机电学校新校区，动工建设湛江幼儿高等师范专科学校、市二技新校区和高技能人才实训基地。加强文化传承保护，促进文化旅游融合发展，提升文化软实力。动工建设湛江文化中心，深化市直文艺团体和经营性文化事业单位改革。加快公立医院改革，开展市管医院与社区卫生服务中心、县（市）医院与乡（镇）卫生院一体化管理体制改革试点，加快乡镇卫生院标准化建设，提高基层卫生医疗机构服务能力和质量。建成湛江中心人民医院新院主体工程和市120医疗急救指挥中心，建设广东医附院海东新院和农垦中心医院门诊大楼。坚持优生优育，完善计生公共服务体系。巩固提升50条基本公共服务均等化示范村建设水平。加快县（市、区）体育场馆建设，做好省运会筹备工作。发展社会福利与慈善事业，建设市残疾人康复中心。推进政府购买社会服务，筹建社会组织孵化服务中心。完成扶贫开发“双到”目标任务。落实军地联席会议制度，推动军民深度融合发展，加强双拥共建、国防教育和民兵预备役工作。抓好第三次经济普查，做好妇女儿童、民族宗教、防震减灾、史志档案、老区、粮食和人防、消防、海防等工作。

　　办好民生实事。在确保完成省政府确定的民生实事基础上，推进实施十大民心工程：推进建设湛江中心人民医院新院、湛江奥体中心、湛江文化中心、职教基地和市级养老服务中心，推进鹤地水库水污染综合整治，推进“五城同创”，推进生态文明区城镇村创建行动，推进村村通自来水工程和村道建设。办成十件民生实事：城乡免费义务教育生均公用经费补助标准小学提高到950元、初中提高到1550元；城乡居民医疗保险年度最高支付限额一档由25万元增至30万元，二档由30万元增至50万元；建设一批市区繁华路段便民交通硬件设施；人均基本公共卫生服务经费标准提高到35元以上；新建平价药店20家、平价商店30家；开工建设保障房3000套、13.5万平方米，建成2000套、9万平方米；改造农村低收入住房困难户住房1万户；建设移民新村85条；完成霞山海滨昌大昌片区排水系统整治；新建9个乡镇（街道）文化站、367个村（社区）文化室。

　　创新社会治理。加快创建“平安湛江”，坚持以群众满意为标准，建立初信初访首问首办和问责考核制度，健全基层综合服务管理平台；坚持对严重暴力和黑恶犯罪“零容忍”，开展打击欺行霸市、农村恶势力和“两抢一盗”、“一清两禁”、反走私等专项行动；新装1万个治安视频监控镜头、新建20个治安卡口；加强普法教育和依法治理工作，健全法律援助制度。强化价格监管，完善价格评估。健全食品原产地追溯和质量标识制度，加大农村卫生室和零售药店监管力度。落实安全生产“党政同责、一岗双责、齐抓共管”制度，落实事故隐患排查整改，规划建设国家级危化品应急救援基地，完善市应急指挥平台，动工建设突发事件预警信息发布中心。

　　各位代表，完成今年目标任务，加强政府建设是保障。我们要按照为民务实清廉的要求，坚持依法依规办事，主动接受人大法律和政协民主监督，加强人大代表建议和政协委员提案办理工作；自觉接受公众和媒体监督。严格按照法定权限和程序行使权力，加强规范性文件、重大行政决策合法性审查，建设法治政府，提高政府科学决策水平。坚持苦干实干创业，树立用结果说话的工作政绩导向，始终把“干”作为一种基本能力，作为解决一切矛盾和问题的有效办法，继续营造“干”的氛围，增强“干”的本领，爱护“干”的干部，在“干”中统一认识，在“干”中解决矛盾，在“干”中推动发展。坚持督查督办落实，着力提升部门执行力，确保政令畅通、令行禁止，确保重点工作快落实、重点项目快建成、重点事项快完成。坚持清正廉洁从政，精心开展党的群众路线教育实践活动，落实中央“八项规定”和省市实施办法，全力解决“四风”突出问题。落实党政机关厉行节约反对浪费条例和国内公务接待管理规定，最大限度压缩行政经费开支。强化行政监察和审计监督，构建监察、司法、审计联动的行政权力监督机制，把权力关进制度的笼子。加强农村集体资金、资产、资源“三资”监管，建设廉洁镇村。主动公开财政预决算、环境保护、社会民生等政务信息，传递湛江发展正能量。全体公务员要秉持公心抓工作、干事业、促发展，做到一身正气、浑身阳光，勤廉双优、德才兼备，树立信念坚定、为民服务、勤政务实、敢于担当、清正廉洁的新时期好干部形象！

　　各位代表，政府工作千头万绪，关键是突出重点抓落实。今年，市政府在全面完成各项目标任务基础上，将着重抓好四件大事：一是把改革当作头等大事，全面深化改革，构建市场化法治化国际化营商环境；二是全力促进中科炼化项目全面开工建设；三是动工建设海西快线，与建设中的海东快线形成海湾两岸两个城市快速通道；四是办好中国海洋经济博览会。各级政府、各部门要围绕发展大局，攻克重点难点，以点带面实现工作新突破、打造工作新亮点、开创工作新局面！

　　各位代表，外界关注湛江，商家看好湛江，广东寄望湛江，人民期待湛江！湛江的机遇是时代的机遇，决不能在我们手中错过！让我们在中共湛江市委的坚强领导下，咬定目标，坚定信心，攻坚克难，扎实苦干，与全市人民一道，用智慧和汗水，谱写湛江发展更加灿烂、更加出彩、更加绚丽的新篇章！
